# Using the teach-back method to improve postpartum maternal-infant health among women with limited maternal health literacy: a randomized controlled study

**DOI:** 10.1186/s12884-022-05302-w

**Published:** 2023-01-09

**Authors:** Gui Zhi Cheng, An Chen, Youdi Xin, Qian Qian Ni

**Affiliations:** 1 The First Affiliated Hospital of University of Science and Technology of China (USTC), Division of Life Sciences and Medicine, University of Science and Technology of China, Hefei, 230001 Anhui China; 2 Institute of Healthcare Engineering, Management and Architecture (HEMA), Department of Industrial Engineering and Management, Aalto University, Maarintie 8, 02150 Espoo, Finland; 3 Department of Obstetrics and Gynaecology, Helsinki University Hospital and University of Helsinki, Haartmaninkatu 2, 00290 Helsinki, Finland; 4 Nordic Healthcare Group Oy, Vattuniemenranta 2, 00210 Helsinki, Finland

**Keywords:** Teach-back, Maternal health literacy, Postpartum maternal-infant health

## Abstract

**Aim:**

This study aimed to evaluate the effects of using the teach-back method among women with limited maternal health literacy (LMHL) on maternal health literacy(MHL), postpartum health behaviours and maternal-infant health outcomes.

**Methods:**

A randomized controlled study was conducted in the obstetrics department of Anhui Provincial Hospital, China. A total of 258 pregnant women with LMHL were recruited at the point of admission to the hospital for birth and randomly assigned to the control group (*n* = 130), where women received routine education sessions, and the teach-back group (*n* = 128), where women received routine education sessions plus a teach-back intervention. The two groups were assessed in terms of MHL before and after the intervention, breastfeeding execution, uptake of 42-day postpartum check-ups, complete uptake of one-time recommended vaccines, and physical health outcomes. Statistical tests were employed for data analysis.

**Results:**

There was no significant difference between the two groups in terms of MHL and other social, demographic, and medical status at baseline. After the intervention, the teach-back group had a higher level of MHL (*p* < 0.001), better postpartum health behaviours in terms of exclusive breastfeeding within 24 hours postpartum (*x*^2^ = 22.853, *p*<0.001), exclusive breastfeeding within 42 days postpartum (*x*^2^ = 47.735, *p*<0.001), uptake of 42-day postpartum check-ups (*x*^2^ = 9.050, *p* = 0.003) and vaccination (*x*^2^ = 5.586, *p* = 0.018) and better maternal-infant health outcomes in terms of the incidence of subinvolution of the uterus (*x*^2^ = 6.499, *p* = 0.011), acute mastitis (*x*^2^ = 4.884, *p* = 0.027), postpartum constipation (*x*^2^ = 5.986, *p* = 0.014), overweight (*x*^2^ = 4.531, *p* = 0.033) and diaper dermatitis (*x*^2^ = 10.896, *p* = 0.001).

**Conclusions:**

This study shows that the teach-back method is effective for enhancing MHL, leading to positive postpartum health behaviours, and improving postpartum maternal-infant health outcomes among women with LMHL. The teach-back method may play an important role in improving postpartum maternal-infant health and could be considered in maternal health education.

**Trial registration number:**

Our trial has been prospectively registered at ClinicalTrials.gov (Ref. No.: NCT04858945) and the enrollment date was 26/04/2021.

**Supplementary Information:**

The online version contains supplementary material available at 10.1186/s12884-022-05302-w.

## Background

The postpartum period (or puerperium), called the “4th trimester”, commonly referring to the time from the expulsion of the placenta through the first six to 8 weeks after birth, is a time of convalescence for the mother, impacts on the mother’s short-term and long-term health and well-being, and lays the foundation for the newborn’s development [1–3]. Most maternal and infant deaths [4, 5], as well as many severe pregnancy- and birth-related complications and child illnesses that may cause lifelong suffering, occur during the postpartum period [6, 7]. Health problems are common among puerperal women. By reviewing relevant studies, Cooklin et al. concluded that approximately 90% of women suffered from at least one physical health problem (e.g., back pain, perineal pain, pain related to caesarean wounds, haemorrhoids, constipation or incontinence) in the first 8 weeks postpartum, over 60% reported two or more physical problems, and between 20 and 80% of puerperal women had breastfeeding problems [8]. According to the World Health Organization (WHO) [9], infections are among the leading causes of newborn deaths, and it is common for newborns to suffer from health problems, e.g., jaundice, which is the most common reason for readmission during the early postpartum period [10]. While maternal and infant health has substantially improved worldwide in recent decades, postpartum-related health issues still need closer attention, especially for mothers and newborns living in low-income and middle-income countries or areas [11, 12]. Considering the epidemiological trend of increasing maternal age, the postpartum period has become more biopsychosocially complex and risky [13]. Promoting overall health and wellbeing for all mothers and infants during the postpartum period, which used to be ignored in many counties, is a rising trend and has been an universal goal included in new national and global health initiatives, strategies, guidelines and recommendations [13–18]. More informed research and evidence-based practices that can prevent postpartum complications and improve puerperal maternal-infant health are needed.

Studies have suggested that birth outcomes and postpartum health could be significantly influenced by maternal health literacy (MHL) [19–22]. MHL, a concept derived from health literacy and proposed by Renkert and Nutbeam, refers to “the cognitive and social skills which determine the motivation and ability of women to gain access to, understand, and use information in ways that promote and maintain their health and that of their children” [23]. Women without an adequate level of MHL are more likely to have problems in perceiving the risks of pregnancy and childbirth, have challenges in interpreting and applying key information provided by health care providers, and be less capable in making reproduction-related decisions [24]. This may lead to negative health behaviours or unhealthy lifestyles and ultimately decrease the mother’s and child’s health statuses. For women during pregnancy and childbirth, especially for the first-time mothers in this life-changing stage, challenges in the acquisition and application of new knowledge and skills for caring for themselves and their child could be demanding and make women stressful [21]. According to the known current studies [25–29], low levels of MHL are quite common among women, and approximately 25–55% of pregnant women have limited health literacy. Mothers with lower incomes, limited resources, low education levels and/or an ethnic minority status usually have lower levels of health literacy [30]. A growing number of studies have shown that limited maternal health literacy (LMHL) is associated with adverse birth outcomes and postpartum health problems, leading to an elevated rate of caesarean section [31], an increased risk of premature birth [32], a higher rate of low birth weight [32], additional risks of postpartum depression [33], a higher risk of maternal overweight [21] and decreased likelihood of breastfeeding [9]. It has also been suggested that LMHL could decrease the likelihood that women have their children immunized [34]. A broad consensus among researchers and practitioners is that better health outcomes for both mothers and children can be achieved by improving MHL [19, 35, 36]. Thus, for improving postpartum maternal and infant health, it is important to provide effective MHL-enhancing support to women, especially to women with LMHL.

It has been widely acknowledged that improvement in health literacy is achievable with appropriate communication strategies, techniques or practices, among which the teach-back method has been considered the most recommendable for its simplicity, harmlessness and effectiveness across different medical settings and populations [37–39]. In health care, the teach-back method is used as a process whereby the health care provider (e.g., nurses and doctors) explains the medical situation, presents relevant information and/or provides advice in a simple and understandable way to the patient, who is then asked to use his or her own words to restate what the health care provider has just told them in a shame-free environment. The health care provider then assesses the patient’s understanding of the subject, identifies misunderstandings, corrects the mistakes, and provides additional information if required; this process continues until the patient can correctly repeat what he or she has just learned and the health care provider confirms the patient’s understanding [38–44]. Studies have found that patient comprehension and recalling of health-related information is limited, as only less than half of the information provided by professionals could be accurately recalled by patients [38, 39, 45, 46]. Patient understanding and memorization of health knowledge and information could be enhanced through these continuous feedback processes. This health literacy-based communication approach has been considered useful in helping patients to better understand their own medical conditions and health information and improving their own abilities to recall and apply medical knowledge in self-care [47, 48]. Studies have shown that the teach-back method is not only effective in improving literacy-related outcomes (e.g., knowledge acquisition, recall and retention) but also helpful in improving patients’ health behaviours (e.g., disease management and self-care behaviours) and health-related outcomes (e.g., hospital readmissions, quality of life and patient satisfaction) [38, 39]. While there has been extensive research on the teach-back method and its use in different clinical areas, with the majority of studies focusing on chronic diseases [38, 39, 49], a limited number of studies have investigated the effects of using the teach-back method in the area of pregnancy and childbirth. Previous studies have focused on certain issues, such as immunization [50], breastfeeding [51], communication [41], and the experience of using the teach-back method [52, 53]. To date, the observation of using teach-back methods for postpartum health is still lacking, and especially, the evidence on the impact of the teach-back method on postpartum health outcomes among women with LMHL is largely missing. Among the known studies, only one with the study context in Iran showed that the teach-back method improved postpartum quality of life [42]. The lack of empirical evidence may hinder the effective use of teach-back based tools and practices for postpartum health. Thus, a context-based investigation on the impacts of using the teach-back method on postpartum health is necessary.

For adding empirical evidence, knowledge and experience of using teach-back method for postpartum health, this study aimed to evaluate the effects of the teach-back method among Chinese women with LMHL in terms of MHL, postpartum health behaviours and postpartum maternal-infant health outcomes. Hefei, the provincial capital of Anhui in China, provides a suitable research context for this subject. Anhui Province, with a population of 61.03 million, had 645,000 newborns in 2020 [54]. According to a provincial survey on the status of MHL in Anhui, only 0.9% of women had adequate health literacy during pregnancy and childbirth [55]. Concerns were raised about maternal and infant health after China introduced a universal two-child policy in 2015, and more worries have been brought up for the recent three-child initiative [56]. Women giving birth are more likely to be multiparous and have advanced maternal age [57]. Considerable effort should be made to ensure maternal-infant health, with special attention paid to women with LMHL.

## Methods

### Ethics approval

This study was approved by the Ethical Committee of the First Affiliated Hospital of University of Science and Technology of China (USTC) (2021-KY104), at Anhui Provincial Hospital, a tertiary care hospital. Our trial has been prospectively registered at ClinicalTrials.gov (Ref. No.: NCT04858945) and the enrollment date was 26/04/2021.

### Study design

This was a randomized controlled trial study with two-arms: a teach-back group and a control group.

### Participants and recruitment

In this randomized controlled trial, the inclusion criteria for participants were as follows: pregnant women who (1) were over 18 years of age; (2) were basically healthy and had not been diagnosed with severe pregnancy complications (e.g.: abruptio placentae, eclampsia); (3) had sufficient commands of communication in Chinese; (4) were between 37^+ 0^ and 41^+ 6^ weeks of pregnancy but had no signs of labour or going into the first stage of labour; (5) had obtained 27 or less points in the baseline measurement of MHL using the Perinatal Maternal Health Literacy Scale (PMHLS) [58]; and had newborns who (1) were born between 37 and 42 weeks of gestation; (2) got a minimum Apgar score of 8 in 5 minutes of being born; and (3) had birth weights between 2500 g and 4000 g. We excluded participants if they (1) had suffered from severe perinatal complications that needed advanced care, e.g., transfer to the intensive care unit, (2) had foetal congenital malformations that were diagnosed during the trial, or (3) did not complete any of the required education sessions planned during the trial.

We recruited women from the Obstetric Department of Anhui Provincial Hospital between 17 May 2021 and 10 August 2021. Two research nurses approached pregnant women on the day they were admitted to hospital for birth with pregnancies that were considered full term (pregnancy weeks between 37^+ 0^ and 41^+ 6^) but did not have any sign of labour or going into the first stage of labour. At our hospital, pregnant women with pregnancies over 37 weeks could admit themselves to wait for labour to start. Before labour started, pregnant women could stay at the hospital ward, got regular check-ups every day and prepared themselves for the coming labour. Our research nurses asked women to complete an online PMHLS questionnaire for the baseline MHL assessment. Eligible pregnant women were informed about the aims, contents, and procedures of the study. Participation was voluntary, and informed consent in written was signed by each participating woman.

## Intervention

### Building a health education team, developing protocols and training staff

Two obstetric care specialists, eight registered nursing supervisors (RNSs) and two master’s-level research nurses comprised a health education team for this study. Obstetric care specialists were primarily responsible for guiding and monitoring the execution of the intervention. Registered supervisor nurses organized education sessions for the participating women and communicated with the women in the education sessions. Research nurses designed the study, collected the data and conducted the analysis, but to avoid critical biases in data analysis and interpretation, they did not participate in any education sessions set in the trial.

Before commencing the trial, a one-day team workshop was organized. The workshop was organized with the following programmes:

1. One research psychologist who was experienced with nurse-patient communication and one education specialist who was experienced in using the teach-back method were invited to introduce the teach-back method to the team.
2. Two research nurses introduced the trial and its process, demonstrated the intervention, and explained the guidelines for communicating with the women in the education sessions. The materials of the education sessions for both teach-back group and control group were developed in accordance with the 55 knowledge and skill items defined in “Maternal and child health literacy - basic knowledge and skills”, a national guidebook issued by the China Ministry of Health in 2012 [59]. The educational materials included power-point presentations, educational video clips, and information booklets. The SHARE Approach, developed by the Agency for Healthcare Research and Quality (AHRQ) [60], was used to form the communication guidelines and protocols to support the education sessions.
3. The research nurses hosted a team discussion to develop work protocols used in the teach-back group and control group and agreed on the contents, timing, processes and communication methods for each education session offered to the participating women. The research psychologist and education specialist commented during the discussion.
4. Teach-back training was organized for 8 RNSs. Laptops and paper copies of the team-agreed education materials, together with communication guidelines and protocols planned to be used during the trial, were distributed to the RNSs. The RNSs were divided into four pairs and practised teach-back education sessions and conventional education sessions in simulated scenarios. The research psychologist, education specialist and two research nurses provided supports to each pair during the training.
5. The workshop closed after one research nurse provided a summary and expressed gratitude to all the participants.

Before the trial started, each RNS was asked to complete a test that consisted of a theoretical examination and practices. The research psychologist, education specialist and two research nurses evaluated each RNS’s performance and gave comments and suggestions for improvements. In the theoretical examination, the RNSs had to answer questions regarding the theory of teach-back and its application. In the practice session, each RNS was asked to communicate with a “patient”, played by a research nurse, and organize one teach-back education session and one conventional education session. Only after passing the test, the RNSs could start to provide education sessions to the women. All RNSs passed the test.

### Education for the teach-back group and control group

During the trial, women from the teach-back group and control group received three education sessions arranged by trained RNSs who played the educator role. The first education session was arranged on the day of admission or the following day before birth. The main purpose of the first session was to help the women understand and prepare for labour. The topics discussed in the first session included birth modes, the labour process and pain relief, newborn health and care (common health issues and basic care skills), perinatal diet and exercises, and perinatal psychological health. On the day of discharge after birth, women received the second group education session hosted by an educator. The main purpose of the second session was to help the women increase their awareness of postpartum health issues, get to know the practice of postpartum recovery and learn newborn care skills. The topics discussed in the second session included postpartum recovery, postpartum maternal health, perineum care, breastfeeding, postpartum blues and depression, neonatal care, neonatal screening tests, newborn vaccinations and early child development. There were three women and one educator attending each group-session. At 2 weeks postpartum, a short online meeting was arranged between one woman and one educator. During the meeting, the educator answered questions raised by the woman and suggested solutions to the difficulties and problems faced by the woman in postpartum recovery and caring for the newborn. The teach-back group and control group shared the educational contents, materials, and the basic communication methods, e.g., power-point presentations, educational video clips, live demonstrations, information booklets, group discussion, and Q&As, except that before the end of each education session, the women in the teach-back group were asked to use their own words to restate what they had just learned, and the educator assessed women’s understanding of the subject, identified misunderstandings, corrected mistakes, and provided additional information if needed, until the women could correctly restate what they were expected to learn. Education sessions offered to the teach-back group lasted longer than the ones offered to the control group. More details about education sessions were presented in Table 1.

**Table 1:** Education sessions for the teach-back group and the control group

Time	Approaches, techniques and tools		Topics	Goals
	Control group	Teach-back Group		
The day or the following day of admission before childbirth	Duration: 45mins Group meeting with three women and one educator; power-point presentation; educational video clips; live demonstration; Q&A	Duration: 60mins Group meeting with three women and one educator; power-point presentation; educational video clips; live demonstration; Q&A; teach-back	Birth mode; labor process and pain relief; newborn health and care (common health issues and care skills); perinatal diet and exercise; perinatal psychological health;	To help women understand and prepare for the labor
The day of discharge	Duration: 60mins Group meeting with three women and one educator; power-point presentation; educational video clips; live demonstration; information booklet; Q&A	Duration: 75mins Group meeting with three women and one educator; power-point presentation; educational video clips; live demonstration; information booklet; Q&A; teach-back	Postpartum recovery (e.g. pelvic floor function recovery and muscle training); postpartum maternal health; perineum care (diet, oral care, perineal care, and hygiene); breastfeeding; postpartum blue and depression; neonatal care (e.g. feeding, skin care, infantile touching, umbilical nursing, and neonatal bathing); neonatal screening tests; newborn vaccination; early child development	To help women understand postpartum health issues, get to know the ways of postpartum recovery and learn newborn care skills
Two-week postpartum	Duration: 20mins Individual online discussion with one woman and one educator Q&A;	Duration: 30mins Individual online discussion with one woman and one educator; Q&A; teach-back	No specific education topic was predefined	To help women address raised questions, difficulties and problems of postpartum recover and caring newborn

### Randomization, participant grouping and educator assigning

We assigned women to study groups by tossing a coin (heads for the teach-back group and tails for the control group). The women were informed about the purpose and main topics of the education sessions, but they were not aware of the forms and techniques used in the trial, nor were they aware if they were assigned to the teach-back group or control group. For the first education session, the women were grouped based on the time of their recruitment, i.e., being the first recruited, being the first grouped and being the first to receive education; for the second education session, the women were grouped based on the time of their discharge, i.e., being the first discharged, being the first grouped and being the first to receive education. The educators were numbered from 1 to 8 according to their work shifts and were assigned to education sessions in sequence and cycle. For the third education session, the educators were assigned to contact women according to their work schedules. A woman might be grouped with different peers in group-education sessions and met with different educators during the trial. In this way, we could reduce the potential influence from educators and group peers.

## Main measures

### The perinatal maternal health literacy scale (PMHLS)

To measure the women’s MHL at baseline (on the day the women were admitted to the hospital) and 42 days postpartum, we employed the Perinatal Maternal Health Literacy Scale (PMHLS) that was developed and validated in 2014 by a research team from Central South University in China [58]. The PMHLS was formed by referring to the 55 knowledge and skill items defined in “Maternal health literacy - basic knowledge and skills”, a national guidebook issued by the China Ministry of Health in 2012 [58], which was also used by this study as the key source to develop the educational contents for both groups. The PMHLS includes a total of 34 items with two response categories (correct =1, incorrect/unknown = 0) and measures three aspects of maternal health literacy, i.e., women’s understanding on the basic knowledge of postpartum health (subscale 1), awareness of basic skills, healthy lifestyles and health behaviours that could be beneficial for postpartum health (subscale 2), and practices of obtaining relevant information from different sources (subscale 3). According to a recently published literature review on the measurement instruments of maternal health literacy [61], PMHLS helps to examine two dimensions of health literacy: functional health literacy that refers to the ability to read and understand health-related information and interactive health literacy that refers to more advanced cognitive and literacy skills to access and apply health-related information [62, 63]. The range of the total score is from 0 to 34, and a higher score indicates a higher level of MHL. The cut-off score is 27, which means that women obtaining a score of 27 points or less are deemed to have low or limited MHL. The Cronbach’s alpha of this measure in our study was 0.824.

### Postpartum health behaviours and maternal-infant health outcomes

This study focused on postpartum health behaviours and maternal-infant health outcomes that have drawn much attention from hospitals and were measured and monitored in routine care. The postpartum health behaviours investigated in this study included 1) whether the mother started exclusive breastfeeding within 24 hours postpartum; 2) whether the mother performed exclusive breastfeeding within 42 days postpartum; and 3) uptake of 42-day postpartum check-ups. The maternal-infant health outcomes included 1) maternal infection occurring within 42 days postpartum; 2) subinvolution at 42 days postpartum; 3) acute mastitis; 4) maternal constipation occurring within 42 days postpartum; 5) overweight (weight before pregnancy subtracted from weight at 42 days postpartum > 20 kg); and 6) diaper dermatitis (DD) within 42 days postpartum.

## Sample size

Based on the previous estimation of LMHL in Anhui (99.1% among pregnant women) [55] and with a hypothesis that the teach-back method would decrease the rate of LMHL from 99.1 to 50%, we estimated that the sample size was 65 women in each group to reach a significance level of 5% with 90% power. We conducted a pilot study with 10 women in each group and calculated sample sizes based on each postpartum health behaviour and maternal-infant health outcome included in this study. The sample size was chosen if it is held over 90% power to detect small effects [64]. EpiCalc2000 (version 1.02) was used to calculate the sample size. Additional File 1 shows the sample calculation for each postpartum health behaviour and maternal-infant health outcome, and the minimum sample size was 81. Considering the 20% drop-out and exclusion rate in follow-up, we decided to recruit at least 102 women for each group.

## Data collection

We collected women’s baseline MHL via an online questionnaire on the day when women were admitted to the hospital. Women were asked to access the online questionnaire via their own electronic devices (smartphones, pads or laptops) and independently complete all questions. Responses with missing answers to the PMHLS questions or submitted in less than 2 minutes or over 30 minutes were considered invalid and excluded. Women with a PMHLS score of 27 or less were asked about their willingness to be included in the study. The women’s social ID number and social-demographic information, including age, ethnic group (Han people/minority), marital status (married/other), education background (primary and below/junior school/high school/college/undergraduate and above), residential area (rural area/county/urban), occupational status (unemployed/employed), monthly family income, insurance type (self-paying/new rural cooperative medical system/worker with medical insurance/other), body mass index (BMI) before pregnancy, parity (unipara/multipara), history of scarred uterus (no/yes), any pregnancy and childbirth-related educational sessions before participating in the current study (no/yes), and number of terminations, were collected from the women via the baseline questionnaire. Information about birth modes (spontaneous labour/caesarean section), postpartum maternal-infant health outcomes and health behaviours was obtained from the hospital’s patient electronic medical record system. When women visited the hospital for a regular 42-day postpartum check-up, their MHL at 42 days postpartum was collected via the same questionnaire used at baseline. Two research nurses checked the quality of the responses. Again, responses with missing answers to the PMHLS questions and those completed in less than 2 minutes or over 30 minutes were considered invalid and excluded.

## Data analysis

One research nurse was responsible for extracting the data and organizing them in the analysis software SPSS 22.0; the other research nurse checked the data. Descriptive statistics were used to quantitatively describe or summarize the basic characteristics of the sample in our study, e.g., means, SDs, frequencies, and medians (*P*_*2*5_, *P*_*75*_). Depending on the nature of the data and the results of normality tests, the Mann–Whitney U test and Wilcoxon paired rank sum test were used to test the differences in MHL scores between the two groups [65]. Comparisons between the groups were made with a conventional chi-square test for categorical variables [66]. A *p* value of 0.05 was set for statistical significance in the analysis.

## Results

### Sample characteristics

**Fig. 1:**
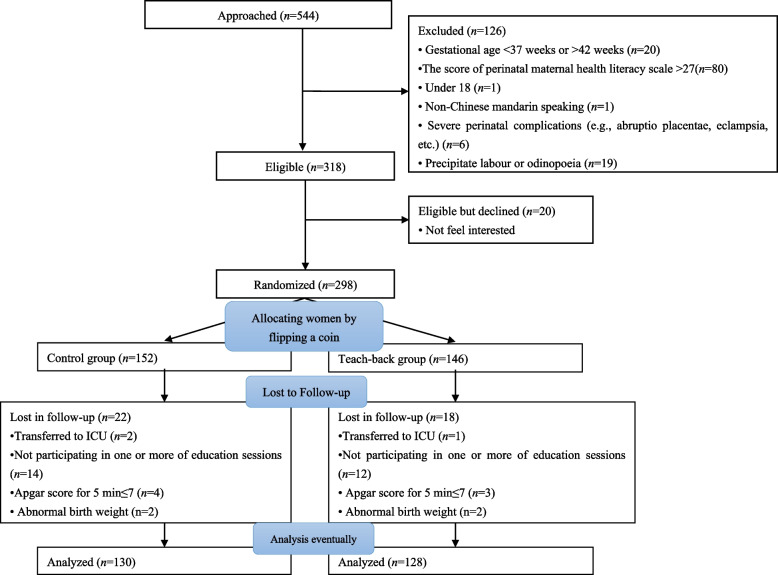
Diagram of recruitment and randomization

Fig. 1 shows the flowchart of the participant recruitment process. In total, 544 pregnant women were approached and screened for MHL, and 298 women (54.78%) were included and allocated to two groups. During the follow-up, 18 participants from the teach-back group and 22 from the control group were excluded for medical reasons. Finally, 128 pregnant women from the teach-back group and 130 from the control group were included in the analysis. Table 2 shows the characteristics of the participants in the two groups. No significant difference was identified between the two groups in terms of age, ethnicity, marital status, education level, residence, occupational status, monthly family income, insurance type, BMI before pregnancy, parity, history of scarred uterus, attendance of any pregnancy and childbirth-related educational sessions before participating in the current study, number of terminations or birth mode.

**Table 2:** Sample characteristics (*n* = 258)

Characteristics	Teach-back group *n* = 128 *n* (%)	Control group *n* = 130 *n* (%) No. (%)	Statistic	*P value*
Age [years, *M* (*P*_25_, *P*_75_)]	29(27.25,33)	32(28,32)	− 0.489^a^	0.625
Ethnic group				
Han people	126(98.4)	126(96.9)	0.651^b^	0.420
Minority	2(1.6)	4(3.1)		
Marital status				
Married	127(99.2)	127(97.7)	0.985^b^	0.321
Other	1(0.8)	3(2.3)		
Education				
Primary and below	1(0.8)	0	−1.261 ^a^	0.207
Junior school	16(12.5)	17(13.1)		
High school	20(15.6)	11(8.5)		
College	82(64.1)	90(69.2)		
Undergraduate and above	9(7.0)	12(9.2)		
Residential area				
Rural area	11(8.6)	16(12.3)	1.551^b^	0.461
County	19(14.8)	23(17.7)		
Urban	98(76.6)	91(70)		
Occupational status				
Unemployed	44(34.4)	41(31.5)	0.235^b^	0.628
Employed	84(65.6)	89(68.5)		
Monthly family income (RMB)				
< 5000	36(28.1)	32(24.6)	−0.054^a^	0.957
~ 8000	46(35.9)	50(38.5)		
~ 15,000	22(17.2)	35(26.9)		
≥15,000	24(18.8)	13(10.0)		
Insurance type				
Private insurance	31(24.2)	20(15.4)	3.400^b^	0.334
New rural cooperative medical system	19(14.8)	21(16.2)		
Occupational health insurance	77(60.2)	87(66.9)		
Other	1(0.8)	2(1.5)		
BMI before pregnancy (mean ± SD)	20.925 ± 2.235	21.257 ± 2.462	1.135^c^	0.258
Normal (18.5 ≤ BMI < 25)	101(78.9)	103(79.2)	0.004^b^	0.949
Abnormal (BMI < 18.5 or BMI ≥ 25)	27(21.1)	27(20.8)		
Parity				
Unipara	77(60.2)	74(56.9)	0.278^b^	0.598
Multipara	51(39.8)	56(43.1)		
History of scarred uterus				
No	100(78.1)	102(78.5)	0.004^b^	0.948
Yes	28(21.9)	28(21.5)		
Experience with any pregnancy and childbirth-related educational sessions before participating in the current study				
No	77(60.2)	82(63.1)	0.233^b^	0.630
Yes	51(39.8)	48(36.9)		
Number of terminations [*M* (*P*_*25*_, *P*_*75*_)]	0(0,1)	0(0,1)	−0.244^a^	0.807
Birth modes				
Spontaneous labor	69(53.9)	66(50.8)	0.254^b^	0.614
Caesarean section	59(46.1)	64(49.2)		

^a^ Mann–Whitney rank test

^b^ Chi-squared test

^c^ *t*-test

### MHL levels of the participants

Table 3 shows the PMHLS scores of the teach-back and control groups at baseline and at 42 days postpartum. At baseline, the two groups had no significant difference in terms of the PMHLS scores (*p* > 0.05) or the scores of the three subscales (*p* > 0.05), but at 42 days postpartum, the two groups had significant differences in the PMHLS scores (*p* < 0.001) and the scores of the three subscales (*p* < 0.001). Half of the women (*n* = 64, 50%) in the teach-back group had the PMHLS scores above 27 at 42 days postpartum, and the percentage was significantly higher than that of the control group (*n* = 11, 8.5%) at 42 days postpartum (*x*^2^ = 53.971, *p* < 0.001).

**Table 3:** PMHLS scores of the teach-back and the control group at baseline and postpartum. (*n* = 258)

PMHLS and subscales	Teach-back group *n* = 128 *M* (*P*_*25*_, *P*_*75*_)	Control group *n* = 130 *M* (*P*_*25*_, *P*_*75*_)	*z* value^b^	*P value*
*Subscale 1: understanding on the basic knowledge of postpartum health*				
Baseline	9(6,11)	9(7,11)	−0.414	0.679
42 days postpartum	12(10,13)	10(8,11.25)	−6.845	<0.001
*z* value^a^	−9.010	−6.977		
*P value*	<0.001	<0.001		
*Subscale 2: awareness of basic skills, healthy lifestyles and health behaviours that could be beneficial for postpartum health*				
Baseline	9(7,11)	9(6.75,10)	−1.147	0.251
42 days postpartum	10(8.25,11)	9(7,10)	−4.310	<0.001
*z* value^a^	−7.188	−4.379		
*P value*	<0.001	<0.001		
*Ssubscale 3: practices of obtaining relevant information from different sources*				
Baseline	2(1,3)	2(1,3)	−1.297	0.195
42 days postpartum	4(3,6)	2(1,3)	−7.148	<0.001
*z* value^a^	−7.809	−3.236		
*P value*	<0.001	<0.001		
*Total score*				
Baseline	20.5(16,24)	20(16,23)	−0.747	0.455
42 days postpartum	26.5(23,29)	21(17,24)	−8.103	<0.001
*z* value^a^	−9.820	−7.073		
*P value*	<0.001	<0.001		

^a^ Wilcoxon Analysis

^b^ Mann–Whitney rank test

At 42 days postpartum, the total PMHLS score of the teach-back group significantly increased from 20.5(16,24) to 26.5(23,29) (*z* = − 9.820, *p* < 0.001), with the score of subscale 1 “understanding on the basic knowledge of postpartum health” increasing from 9(6,11) to 12(10,13) (*z* = − 9.010, *p* < 0.001), the score of subscale 2 “awareness of basic skills, healthy lifestyles and health behaviours that could be beneficial for postpartum health” increasing from 9(7,11) to 10(8.25,11) (*z* = − 7.188, *p* < 0.001), and the score of subscale 3 “practices of obtaining relevant information from different sources” increasing from 2(1,3) to 4(3,6) (*z* = − 7.809, *P* < 0.001). The total PMHLS score of the control group significantly increased from 20(16,23) to 21(17,24) (*z* = − 7.073, *p* < 0.001), with the score of subscale 1 increasing from 9(7,11) to 10(8,11.25) (*z* = − 6.977, *p* < 0.001), the score of subscale 2 increasing from 9(6.75,10) to 9(7,10) (*z* = − 4.379, *p* < 0.001), and the score of subscale 3 increasing from 2(1,3) to 2(1,3) (*z* = − 3.236, *p* < 0.001).

### Comparison of postpartum health behaviours and maternal-infant health outcomes between the two groups

Compared to the control group, the teach-back group had significantly better postpartum health behaviours in terms of exclusive breastfeeding within 24 hours postpartum (*x*^2^ = 22.853, *p*<0.001), exclusive breastfeeding within 42 days postpartum (*x*^2^ = 47.735, *p*<0.001) and uptake at the 42 days postpartum check-up (*x*^2^ = 9.050, *p* = 0.003) (Table 4). Regarding maternal-infant health outcomes, the incidences of subinvolution of the uterus (*x*^2^ = 6.499, *p* = 0.011), acute mastitis (*x*^2^ = 4.884, *p* = 0.027), postpartum constipation (*x*^2^ = 5.986, *p* = 0.014), overweight (*x*^2^ = 4.531, *p* = 0.033) and diaper dermatitis (*x*^2^ = 10.896, *p* = 0.001) were significantly lower in the teach-back group. However, there was no significant difference between the two groups in the rate of postpartum infection (*x*^2^ = 2.239, *p* = 0.135). During the study, practitioners working at community-level maternal and child clinics suggested that infant vaccination could be an important outcome reflecting maternal-infant health behaviours. We included the complete uptake of recommended vaccines (i.e., receiving all vaccines that were recommended in national and provincial vaccination programmes in a timely manner) as an additional postpartum health outcome indicator. The results showed that the teach-back group had better performance than the control group in terms of vaccination (*x*^2^ = 5.586, *p* = 0.018). The statistical power of the analysis on vaccination behaviours reached an acceptable level, which was 95.08% (Additional File 2).

**Table 4:** Comparison of postpartum health behaviours and maternal-infant health outcomes between the teach-back group and the control group (*n* = 258)

Teach-back group *n* = 128 *n* (%)		Control group *n* = 130 *n* (%)	*x*^2^	*P value*
*Postpartum health behaviours*				
Exclusive breastfeeding within 24 hours postpartum	72(56.3)	35(26.9)	22.853 ^b^	<0.001
Exclusive breastfeeding within 42 days postpartum	75(58.6)	22(16.9)	47.735 ^b^	<0.001
Uptake of check-up at the 42 days postpartum	122(95.3)	109(83.8)	9.050 ^b^	0.003
Complete uptake of recommended vaccines	125(97.7)	118(90.8)	5.586 ^b^	0.018
*Maternal-infant health outcomes*				
Postpartum infection	0	4(3.1)	2.239 ^a^	0.135
Subinvolution of uterus	3(2.3)	13(10)	6.499 ^b^	0.011
Acute mastitis	25(19.5)	41(31.5)	4.884 ^b^	0.027
Postpartum constipation	32(25)	51(39.2)	5.986 ^b^	0.014
Overweight	23(18)	38(29.2)	4.531 ^b^	0.033
Diaper dermatitis	19(14.8)	42(32.3)	10.896 ^b^	0.001

^a^ Continuity correction

^b^ Pearson chi-squared test

## Discussion

### Interpretations of the main findings

This study shows that the teach-back method is an effective method for enhancing MHL, leading to positive postpartum health behaviours, and improving postpartum maternal-infant health outcomes among women with LMHL.

In this study, the education programme embedded in the teach-back method was more effective than the conventional one in improving MHL among women with LMHL. This result is consistent with those from the majority of studies on the use and effects of the teach-back method in maternal and infant care as well as in other clinical settings [38, 39]. This provides additional evidence that the teach-back method can benefit patients with LMHL and might be a valuable addition to patient education in different health care settings [38, 39, 43, 44].

Previous studies have not shown consistent results regarding the effects of the teach-back method in promoting self-care skills, healthy lifestyles, and health behaviours [49]. This RCT presents that the teach-back method had positive effects on positive postpartum health behaviours, i.e., increasing the likelihood of exclusive breastfeeding within 24 hours postpartum, exclusive breastfeeding within 42 days postpartum, uptake of 42-day postpartum check-ups, and complete uptake of recommended vaccines among women with LMHL. Kaufman et al. found that women with LMHL were less likely to exclusively breastfeed [67]. The results of our study suggest that the teach-back method could be useful in promoting breastfeeding among women with LMHL. The potential positive effect of the teach-back method on breastfeeding was also revealed by a recent published study that applied teach-back as one key element in a postpartum breastfeeding support program to increase the rate of exclusive breastfeeding during early postpartum period [51]. Similar to our study, Wilson et al. found that families who received teach-back education were more likely to receive a full course of the hepatitis B vaccination for their children than those who received conventional education [50]. This indicates that the teach-back method could be an effective method for promoting immunization programmes and increasing vaccination among newborns.

The present study shows that the incidences of postpartum maternal-infant complications and health problems, including subinvolution of the uterus, acute mastitis, constipation, overweight and diaper dermatitis, were significantly reduced by the teach-back education programme that was provided to women with LMHL. This result aligns with previous studies that have agreed on the positive effects of this educational method on health outcomes in different clinical settings [39, 68, 69], including Ghiasvand et al., who performed a study exploring the effect of the teach-back method on postpartum quality of life. Observing the significant effects of the teach-back method on improved postpartum physical health, this study shows that the teach-back method, by improving women’s awareness and knowledge of postpartum complications and increasing their understanding of how to deal with postpartum health problems, could be useful in preventing postpartum complications and other health problems [42]. Our investigation found no significant difference in the rate of postpartum infection (*x*^2^ = 2.239, *p* = 0.135) between the two groups, which was probably due to the fact that the incidence of postpartum infection in our sample was low, while it is approximately 2.33% in China [70].

## Implications

As health care providers are aiming to reduce the length of postpartum hospital stay, women’s health literacy and self-care competence are becoming increasingly important, which requires effective strategies that can support women not only in understanding complex health information but also in applying this information in everyday life to improve self-care or care skills, build healthy lifestyles and improve health status. Our study supports the use of the teach-back method in perinatal care for women with LMHL to enhance their MHL, leading to positive postpartum health behaviours and improving postpartum maternal-infant health outcomes.

While the mode of delivering teach back has been well defined, good strategies and practices of implementing it in care routines have not been well recognized, and there is little guidance for successfully embedding the teach-back method in different settings [38]. In this study, the structure and timing of teach-back embedded in education programs might be important for its effectiveness. Starting from late pregnancy, women with LMHL received three education sessions. According to previous studies, e.g., Amoah et al. and Fei et al., during the last trimester of pregnancy, women may have a stronger motivation for and be more proactive in learning information and knowledge that could support them in exercising postpartum self-care or self-management, preventing postpartum complications and promoting maternal and child health [71, 72].

## Contributions

This study makes up for the shortage and lack of research-based evidence about the use of the teach-back method for postpartum maternal and infant health by investigating the effects of the teach-back methods in terms of MHL, postpartum health behaviours, and postpartum maternal-infant health outcomes. This study provides tangible knowledge and practical experience for successfully implementing the teach-back method in routine practice. The knowledge and insights produced by this study could help health professionals in maternal and infant care or other health care settings design and implement education programs incorporated with the teach-back technique for promoting patient health.

### Strengths, limitations and future study

This study was well designed and rigorously executed, following RCT standards. The intervention, i.e., teach-back embedded in education programmes targeting women with LMHL, and education protocols for the two groups were systematically and contextually developed, following the national guidebook of improving MHL and commonly agreed communication guidelines. Nurses who provided education sessions to the women were invited to participate in the workshop for developing the intervention and protocols, so that nurses could develop a deep understanding on the intervention and an agreement on the implementation of the trial could be achieved among nurses. The nurses were intensively trained, and their performances were evaluated by experts before the trial. In this study, the factors that might affect the results of the trial were well considered. For the baseline analysis, this study collected information about whether women had previous experience with pregnancy and childbirth-related education sessions, which was missing in the majority of relevant studies [50].

This study has some limitations. First, due to the nature of the trial, a nurse who provided education sessions to the women was not blinded to the structure and contents of the education sessions to which she was assigned. Second, the practice of grouping women for the first two education sessions may result in within-group interactions or other intra-cluster effects, which could bias the results, although we took some efforts to reduce the bias. Third, caution should be placed on the generalizability of this study, which was conducted in a tertiary hospital located in a moderately developed region. Further studies should explore the effects of the presented intervention in various contexts. Fourth, the cost of using this teach-back embedded education programme (e.g., staff training, workloads for nurses, resource additions, and organizational changes) has not been investigated, so large-scale implementation of this method in clinical routine is still uncertain. Cost-effectiveness analysis and user experiences should be included in future research. Fifth, this study did not provide solid evidence of the retention and the long-term effects of using the teach-back method on maternal and child health, which requires further exploration. The teach-back supported education programme should be further developed based on scientific research-based results.

## Conclusion

This RCT presents evidence that the teach-back method is an effective approach to enhancing MHL levels, changing health behaviours, and improving postpartum maternal-infant health outcomes among women with LMHL. The teach-back method could play an important role in improving postpartum maternal-infant health and should be considered in health education targeting pregnant and postpartum women with LMHL. However, research on its cost-effectiveness, long-term impacts, and user experiences is warranted.

## Supplementary Information

**Additional file 1.** Sample size calculation.

**Additional file 2:.** statistical power of analysis on vaccination.

## Data Availability

The dataset generated and analysed for this study is not publicly available due to the restrictions claimed in the document of the research permission and ethical approval. But the data are available from the ethics committee of the First Affiliated Hospital Ethics Committee of USTC for researchers who meet the criteria for access to confidential data. To request access to the data, please contact the ethics committee of the First Affiliated Hospital Ethics Committee of USTC or the main researcher Qianqian Ni.

## Abbreviations

LMHL: Limited Maternal Health Literacy
MHL: Maternal Health Literacy
PMHLS: Perinatal Maternal Health Literacy Scale
WHO: World Health Organization
RNSs: Registered Nursing Supervisors
AHRQ: Agency for Healthcare Research and Quality
DD: Diaper dermatitis
BMI: Body Mass Index
