# Supplementary material for: Using the teach-back method to improve postpartum maternal-infant health among women with limited maternal health literacy: a randomized controlled study
Source: BMC Pregnancy Childbirth. 2023 Jan 9;23:13. doi: 10.1186/s12884-022-05302-w (PMC9827634; doi:10.1186/s12884-022-05302-w)
Supplement: Supplementary file 1 — Additional file 1. Sample size calculation. [file 12884_2022_5302_MOESM1_ESM.docx]

**Additional file 1** Sample size calculation

**The file shows the sample calculation for each postpartum health behaviour and maternal-infant health outcome.**

**Exclusive breastfeeding within 24 hours postpartum**

Sample - Size - Two rates

Rate 1 : 0.20

Rate 2 : 0.50

Significance : 0.05

Power : 90%

Sample size : 81 (each group)

: 162 (overall)

**Exclusive breastfeeding within 42 days postpartum**

Rate 1 : 0.60

Rate 2 : 0.20

Significance : 0.05

Power : 90%

Sample size : 52 (each group)

: 104 (overall)

**Uptake of 42-day postpartum check-ups**

Sample - Size - Two rates

Rate 1 : 1.00

Rate 2 : 0.50

Significance : 0.05

Power : 90%

Sample size : 62 (each group)

: 124 (overall)

**Maternal infection**

Sample - Size - Two rates

Rate 1 : 0.00

Rate 2 : 0.20

Significance : 0.05

Power : 90%

Sample size : 52 (each group)

: 104 (overall)

**Subinvolution of uterus**

Sample - Size - Two rates

Rate 1 : 0.00

Rate 2 : 0.20

Significance : 0.05

Power : 90%

Sample size : 52 (each group)

: 104 (overall)

**Acute mastitis**

Sample - Size - Two rates

Rate 1 : 0.00

Rate 2 : 0.30

Significance : 0.05

Power : 90%

Sample size : 34 (each group)

: 68 (overall)

**Postpartum constipation**

Sample - Size - Two rates

Rate 1 : 0.10

Rate 2 : 0.40

Significance : 0.05

Power : 90%

Sample size : 58 (each group)

: 116 (overall)

**Overweight**

Sample - Size - Two rates

Rate 1 : 0.10

Rate 2 : 0.40

Significance : 0.05

Power : 90%

Sample size : 58 (each group)

: 116 (overall)

**Diaper dermatitis**

Sample - Size - Two rates

Rate 1 : 0.10

Rate 2 : 0.40

Significance : 0.05

Power : 90%

Sample size : 58 (each group)

: 116 (overall)
