# Supplementary material for: Using the teach-back method to improve postpartum maternal-infant health among women with limited maternal health literacy: a randomized controlled study
Source: BMC Pregnancy Childbirth. 2023 Jan 9;23:13. doi: 10.1186/s12884-022-05302-w (PMC9827634; doi:10.1186/s12884-022-05302-w)
Supplement: Supplementary file 2 — Additional file 2:. statistical power of analysis on vaccination. [file 12884_2022_5302_MOESM2_ESM.docx]

**Additional file 2: statistical power of analysis on vaccination**

# **χ² tests on vaccination** **behaviour (teach-back group vs control group)**

**χ² tests -** Goodness-of-fit tests: Contingency tables

**Analysis:** A priori: Compute required sample size

**Input:** Effect size w = 0.3

α err prob = 0.05

Power (1-β err prob) = 0.95

Df = 1

**Output:** Noncentrality parameter λ = 13.0500000

Critical χ² = 3.8414588

Total sample size = 145

Actual power = 0.9507851
